# A simple yet efficient approach for electrokinetic mixing of viscoelastic fluids in a straight microchannel

**DOI:** 10.1038/s41598-022-06202-x

**Published:** 2022-02-14

**Authors:** C. Sasmal

**Affiliations:** Soft Matter Engineering and Microfluidics Lab, Department of Chemical Engineering, Indian Institute of Technology Ropar, Rupnagar, Punjab 140001 India

**Keywords:** Chemical engineering, Computational science

## Abstract

Many complex fluids such as emulsions, suspensions, biofluids, etc., are routinely encountered in many micro and nanoscale systems. These fluids exhibit non-Newtonian viscoelastic behaviour instead of showing simple Newtonian one. It is often needed to mix such viscoelastic fluids in small-scale micro-systems for further processing and analysis which is often achieved by the application of an external electric field and/or using the electroosmotic flow phenomena. This study proposes a very simple yet efficient  strategy to mix such viscoelastic fluids based on extensive numerical simulations. Our proposed setup consists of a straight microchannel with small patches of constant wall zeta potential, which are present on both the top and bottom walls of the microchannel. This heterogeneous zeta potential on the microchannel wall generates local electro-elastic instability and electro-elastic turbulence once the Weissenberg number exceeds a critical value. These instabilities and turbulence, driven by the interaction between the elastic stresses and the streamline curvature present in the system, ultimately lead to a chaotic and unstable flow field, thereby facilitating the mixing of such viscoelastic fluids. In particular, based on our proposed approach, we show how one can use the rheological properties of fluids and associated fluid-mechanical phenomena for their efficient mixing even in a straight microchannel.

## Introduction

A wide variety of complex fluids such as polymer solutions, emulsions, suspensions, foams, etc., are frequently used in various microfluidic applications^1,2^. Additionally, various biofluids, such as blood, saliva, cerebrospinal fluid, DNA and protein suspensions, etc., are also routinely used in microfluidic and nanofluidic processes, for instance, diagnostics and biochemical analyses. These fluids, very often, do not show the simple Newtonian behaviour (which is described by the well-known Newton’s law of viscosity), but show various complex non-Newtonian behaviour, for example, shear-thinning, shear-thickening, yield stress, etc. Apart from these, many experimental investigations have also found that most of these fluids exhibit a great extent of viscoelastic properties^3–7^. The transportation as well as mixing of such fluids is often needed in various small-scale micro and nanofluidic systems for further processing and analysis^8^. In doing so, an electroosmotic flow (EOF) technique is often used wherein the fluid motion is created due to the interaction between the electrical double layer (EDL) developed along a charged surface and an externally applied electric field^9^. From the past several decades, a significant body of literature, comprising of both theoretical (analytical and numerical) and experimental investigations, is present on how different factors, such as wall zeta potential, applied voltage, patterned surface, rotating or non-rotating surface, system size and structure, electric field type, i.e., AC or DC, presence of obstacles, etc., can influence this electrokinetic transport of various simple Newtonian as well as complex non-Newtonian fluids^10–16^.

The flow dynamics during the electrokinetic transport of complex fluids, particularly for viscoelastic fluids, is found to be much more complex and rich in physics than that seen either for simple Newtonian or non-Newtonian generalized Newtonian fluids (GNFs). For instance, some numerical and experimental studies have found the existence of an instability, known as the electro-elastic instability (EEI), in electrokinetic flows of viscoelastic fluids in various systems. These instabilities are associated with an unstable and fluctuating flow field and originated once the Weissenberg number, *Wi*, (which is a dimensionless number defined as the product of the fluid relaxation time $\lambda$ and the characteristic strain rate ${\dot{\gamma }}$. It signifies the relative importance of the elastic and viscous forces) exceeds a critical value. This critical value depends on many factors such as geometry, rheology of working fluids, external forces to induce the flow field, etc. For example, Afonso et al.^17^ was probably the first who found the existence of these electro-elastic instabilities in their numerical simulations of electrokinetic flows of viscoelastic fluids in a cross-slot micro-geometry. Later, Pimenta and Alves^18^ experimentally verified the existence of these instabilities for the same geometry. Recently, Sadek et al.^19^ and Ji et al.^20^ also observed these kinds of instabilities during the flow through a contraction and expansion micro-geometry in their experimental and numerical analysis, respectively. Only recently, Datta et al.^21^ presented an extensive discussion and review on the origin, mechanism and properties of these elastic instabilities originated in various systems ranging from simple cross-slot geometry to complex porous media. They discussed both the experimental and numerical perspectives of these elastic instabilities and provided the future scope for studying and applying them in many practical applications in detail. Although, the studies reviewed by Datta et al. are based on pressure-driven flows; however, the origin and mechanism of these elastic instabilities should be independent of the external agency for driving the flow, i.e., whether it is pressure-driven or electrokinetic-driven. The number of corresponding studies on these elastic instabilities in electrokinetic flows is very limited as compared to that available for pressure-driven flows. Also, note that these instabilities are different from that of electrokinetic instability (EKI)^22^, which is also often seen during the electrokinetic flows. The former one is originated in viscoelastic fluids due to the interaction between the elastic stresses generated in the system and the presence of streamline curvature in the system^23,24^, whereas the latter one is generally developed in Newtonian fluids due to the presence of electrical conductivity gradient^25–27^.

After the onset of this elastic instability, as the Weissenberg number further increases, the unstable flow field ultimately transits to a more chaotic and turbulent-like flow state. This is known as the elastic turbulence (ET) regime, which is already seen in many pressure-driven flows^28–31^. The origin of this elastic turbulence is totally different from that of regular hydrodynamic turbulence. While the former is originated due to the presence of elastic stresses and in the presence of negligible inertial forces (mainly due to the small-scale of these systems), the latter is established due to the instability driven by the inertial forces. However, these two types of turbulence show some similarities in their spatio-temporal fluctuation hydrodynamics^28^. Therefore, likewise the regular hydrodynamic turbulence, one can also expect that the elastic turbulence will increase the mixing or the rate of transport processes. This is, indeed, found in many recent studies^32–34^. However, all of these studies were conducted for pressure-driven flows, and there is no corresponding study available for electrokinetic flows. It is imperative to investigate as the mixing and heat/mass transfer rate enhancement in micro-scale systems is often a challenging task regardless of the external driving agency (pressure or electric field) due to the existence of the laminar flow condition. Therefore, over the years, many designs and techniques based on both passive and active modes have been developed to enhance the mixing efficiency in various micro-scale systems; for instance, see some excellent review articles present on the same in the literature^35,36^. In this study, we aim to propose a simple yet efficient strategy for the mixing of complex viscoelastic fluids based on the numerical investigation. In particular, we aim to show how the mixing process of these complex fluids can be achieved even in a straight microchannel based on our proposed technique by using the phenomena of electro-elastic instability and electro-elastic turbulence.

## Proposed approach and governing equations

**Figure 1:**
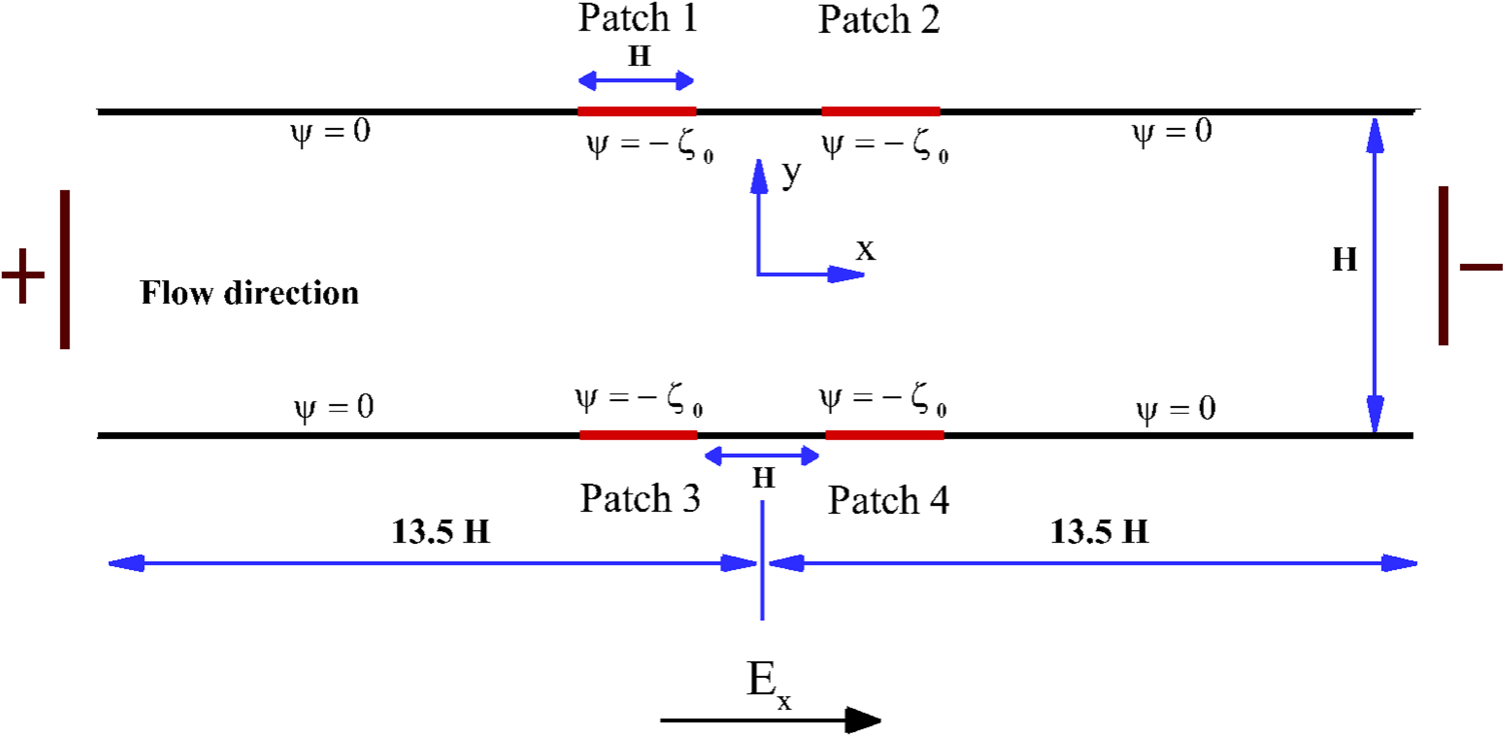
Schematic of the proposed setup for efficient electrokinetic mixing of viscoelastic fluids.

As mentioned in the preceding section, in this study, we aim to propose a simple yet efficient strategy for mixing viscoelastic fluids in a straight microchannel. The schematic of the proposed setup is shown in Fig. 1. It consists of a straight microchannel of the height of $H (= 50 \,\mu m )$ and of the total length of 27*H*. The microchannel has four patches on the surface: two on the top surface (Patch 1 and Patch 2) and two on the bottom surface (Patch 3 and Patch 4). These patches have a constant negative wall zeta potential of $\zeta _{0}$, as schematically shown in Fig. 1. The rest of the microchannel surface possesses a zero wall zeta potential. We have placed two electrodes at the channel inlet and outlet, and an external potential bias and/or voltage $V_{0}$ is applied between the two electrodes. This, in turn, generates an electric field strength $E_{x} = \frac{V_{0}}{L}$ between the two electrodes, where *L* is the total distance between the inlet and outlet of the microchannel. The electroosmotic flow will happen from the anode towards the cathode due to the interaction between the external applied electric field and the net charges accumulated within the EDL that is formed in the proximity of the patches on the microchannel surface. To facilitate this electrokinetic flow, the viscoelastic fluid should be mixed with a binary monovalent electrolyte, for example, KCl or NaCl. The fluid is assumed to be incompressible in nature. The flow field induced by the electric field will be governed by the following equations, namely,

Continuity equation:

1 $$\begin{aligned} \nabla \cdot \mathbf{u } = 0 \end{aligned}$$

Cauchy momentum equation:

2 $$\begin{aligned} \rho \left( \frac{\partial \mathbf{u }}{\partial t} + \mathbf{u } \cdot \nabla \mathbf{u } \right) = - \nabla p + \eta _{s} \nabla ^2 \mathbf{u } + \nabla \cdot \varvec{\tau }_{p} - \rho _{e} \Phi _{ext} \end{aligned}$$

In the above equation, $\varvec{u}$ is the velocity vector, *t* is the time, *p* is the pressure, $\eta _{s}$ is the solvent viscosity, $\rho _{e}$ is the charge density, $\Phi _{ext}$ is the potential originated due to the application of an external electric field and $\varvec{\tau }_p$ is the extra stress due to the presence of viscoelastic microstructure like polymer molecules. Various relations for the evaluation of $\varvec{\tau }_p$ are present in the literature depending upon the type of the viscoelastic constitutive equation. In the present study, we have used the Oldroyd-B viscoelastic constitutive equation to evaluate $\varvec{\tau }_p$. This particular viscoelastic constitutive equation mimics the rheological behaviour of a constant shear viscosity viscoelastic fluid or the so-called Boger fluid^37^. This model was derived based on the kinetic theory of polymers in which a polymer molecule is assumed to be a dumbbell with two beads connected by an infinitely stretchable elastic spring^38^. According to this model, the polymeric stress components are evaluated as follows^38,39^

3 $$\begin{aligned} \varvec{\tau }_p = \frac{\eta _{p}}{\lambda } \left( \varvec{A} - \varvec{I} \right) \end{aligned}$$

where $\eta _{p}$ is the polymer viscosity, $\lambda$ is the polymer relaxation time, $\varvec{A}$ is the polymeric conformation tensor and $\varvec{I}$ is the identity tensor. The evaluation equation for the conformation tensor for an Oldroyd-B fluid is given as

4 $$\begin{aligned} \frac{\partial \varvec{A}}{\partial t} + \varvec{u} \cdot \nabla \varvec{A} = \varvec{A} \cdot \nabla \varvec{u} + \left( \nabla \varvec{u} \right) ^{T} \cdot \varvec{A} - \frac{1}{\lambda } \left( \varvec{A} - \varvec{I} \right) \end{aligned}$$

The total electric potential $(\Psi )$ in the system is computed by solving the Gauss’s law as follows

5 $$\begin{aligned} \nabla \cdot \left( \varepsilon \nabla \Psi \right) = \rho _{e} \end{aligned}$$

The charge density is calculated as $\rho _{e} = F \sum _{i=1}^{N} z_{i} c_{i}$ where *F* is the Faraday’s constant (96485.33289 C $\cdot$ mol$^{-1}$), $z_{i}$ is the charge valence on species *i* and $c_{i}$ is the concentration of species *i*. The electric field is calculated as $\varvec{E} = -\nabla \Psi$. Furthermore, the total electric potential in the system is decomposed into two components, namely, one originating due to the externally applied electric field $(\Phi _{ext})$ and the other arising due to the presence of charge on the microchannel walls $(\psi )$, i.e., $\Psi = \Phi _{ext} + \psi$. After the decomposition, the following equations are solved to get the potential distribution in the system

6 $$\begin{aligned} \nabla \cdot \left( \varepsilon \nabla \psi \right) = \rho _{e} \end{aligned}$$

7 $$\begin{aligned} \nabla \cdot \left( \varepsilon \nabla \Phi _{ext} \right) = 0 \end{aligned}$$

In the present study, the thickness of the electric double layer (EDL) formed along the microchannel wall is much smaller than the height of the microchannel, and hence we have used the Poisson-Boltzmann (PB) equation to calculate the ion distribution in the system. Under this assumption, the Gauss’s law becomes

8 $$\begin{aligned} \nabla \cdot \left( \varepsilon \nabla \psi \right) = F \sum _{i = 1}^{N} z_{i} c_{i,0}\, exp \left( - \frac{e z_{i}}{k T} \psi \right) \end{aligned}$$

where $c_{i,0}$ is the bulk concentration of ion species *i* in the system, *e* is the electron charge (1.6021766341$\times$10$^{-9}$ C), *k* is the Boltzmann’s constant (1.380649$\times$10$^{-23}$ J$\cdot$K$^{-1}$) and *T* is the absolute temperature of the fluid. The present study has been carried out for the following values of these parameters: $c_{i,0} = 9.44 \times 10^{-5}$ mol/m$^3$, T = 298 K, $\varepsilon = 7.0922 \times 10^{-10}$ F/m, $\zeta _{0} = 20$ mV, $E_{x} = 10000$ V/m. In order to quantify the viscoelasticity of the complex fluid, we have used the Weissenberg number defined as $\frac{\lambda U_{0}}{H}$. In the present study, the value of this number is varied in between 0 and 3. This range of the Weissenberg number is physically justified under the current conditions for various viscoelastic fluids such as polyethylene oxide (PEO), polyacrylamide, etc^40,41^. Here $U_{0}$ is the Helmholtz-Smoluchowski velocity^9^ defined as $\frac{\varepsilon \zeta _{0} E_{x}}{\eta _{0}}$, where $\eta _{0}$ (= 0.0015 Pa$\cdot$s) is the zero-shear rate viscosity of the complex fluid. This study has simulated a perfect creeping flow condition (i.e., $Re = 0$) by setting the inertial terms of the momentum equation equal to zero. 

Dye transport equation: The following convective-diffusive equation has been solved to track the dye concentration inside the microchannel in the case of evaluation of the mixing phenomena.

9 $$\begin{aligned} \frac{\partial c}{\partial t} + \varvec{u} \cdot \nabla c = D \nabla ^{2} c \end{aligned}$$

In the above equation, *c* is the dye concentration and *D* is the diffusivity of the dye. In this study, the Peclet number $(Pe = \frac{H U_{0}}{D})$ is chosen much larger than one so that the mixing phenomena of dye due to the convection becomes dominant over due to the diffusion.

## Results

**Figure 2:**
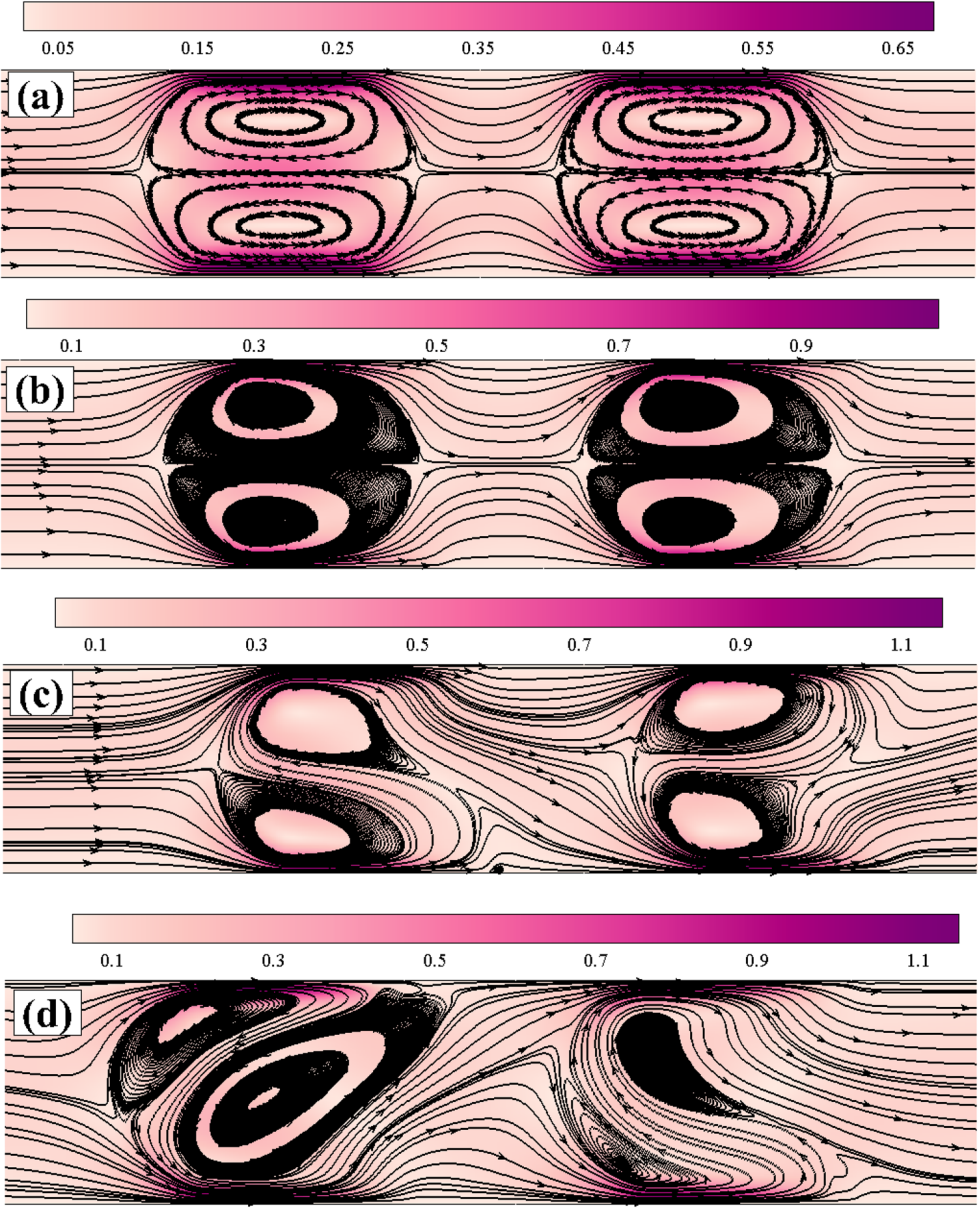
Representative streamlines and velocity magnitude plots both for Newtonian (**a**) and viscoelastic fluids at (**b**) Wi = 1 (**c**) Wi = 2 (**d**) Wi = 3.

After solving the aforementioned governing equations based on the open-source CFD code OpenFOAM (the details are provided in the Methods section), we now discuss the corresponding flow and mixing phenomena results. First, we present the results on the flow dynamics inside the microchannel. The results for the simple Newtonian fluids are also considered in this study. This is to show how the flow and mixing phenomena can become complex for viscoelastic fluids in comparison to that seen for Newtonian fluids under otherwise identical conditions. Figure 2 shows the streamlines and velocity magnitude plots inside the microchannel both for Newtonian and viscoelastic fluids. For simple Newtonian fluids (sub-Fig. 2a), two vortices (namely, one rotating clockwise and another rotating counter-clockwise formed near the upper and lower halves of the channel, respectively) are seen to present between the region of patches 1 and 3. The size and shape of these two vortices are exactly the same. Similarly, two vortices with the same shape and size are also formed between the region of patches 2 and 4. Therefore, a perfect symmetry (along both the horizontal and vertical mid-planes passing through the origin of the microchannel) in the flow field can be seen for Newtonian fluids, which is expected for such fluids under the creeping flow condition. The velocity magnitude is seen to be high near the regions where the patches are present. This is due to the formation of EDL in these regions, which eventually drives the flow from the left-hand side towards the right-hand side of the microchannel. The regions between the two vortices become highly extensional in nature because of their rotation in clockwise and counter-clockwise directions, resulting in the formation of a high-velocity magnitude zone in these regions also.

On the other hand, for viscoelastic fluids, a similar kind of trend in the flow profile is observed as that seen for Newtonian fluids at low values of the Weissenberg number (results are not shown here). This is mainly because of the presence of low elastic stresses at these low values of the Weissenberg number. However, as the Weissenberg number gradually increases to higher values, the flow dynamics inside the microchannel progressively becomes complex. For instance, at $Wi = 1$ (Fig. 2b), the vortices formed between the two patches become more concentrated and shorter in size than that seen for Newtonian and viscoelastic fluids at low Weissenberg numbers. Furthermore, the vortices lost the symmetry in their shape along the vertical mid-plane passing through their centers. Therefore, the symmetry in the flow profile breaks down as the Weissenberg number gradually increases. All these happen due to the increase in the elastic forces with the Weissenberg number, which have a tendency to suppress the vortex formation. Once again, high-velocity magnitude zones are formed near the patches and between the regions of two vortices at y = 0, as observed for Newtonian and viscoelastic fluids at low Weissenberg numbers. Up to this value of the Weissenberg number for viscoelastic fluids and also for Newtonian fluids (for which the Weissenberg number is essentially zero), the streamlines present either in the upper half or lower half of the microchannel never cross the horizontal plane present at y = 0. Therefore, one can expect no mixing of fluids (if two viscoelastic fluids are present in the upper and lower halves of the microchannel) up to this value of the Weissenberg number.

**Figure 3:**
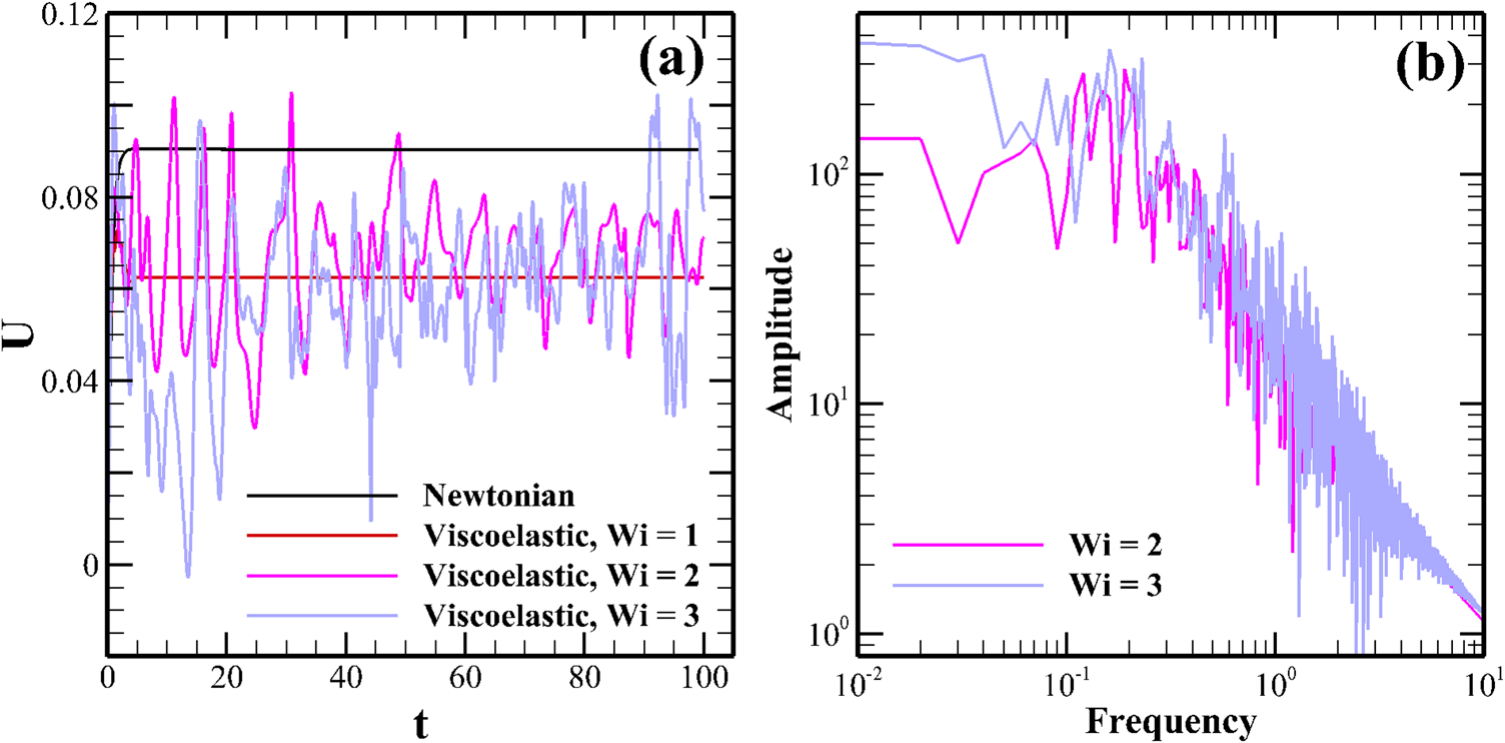
(**a**) Temporal variation of the stream-wise velocity at a probe location placed at the origin (**b**) The corresponding power spectral density plot of the velocity fluctuations for viscoelastic fluids at two different Weissenberg numbers, namely, 2 and 3.

As the Weissenberg number further increases to 2, the regular shape (kidney-like shape) of vortices is now totally destroyed, and their size and shape become unequal and irregular from each other, see Fig. 2c. Most importantly, one can see that the streamlines now cross the horizontal mid-plane passing through the origin of the channel. This could facilitate mixing, which will be discussed later in this section. All these suggest that the flow field inside the microchannel becomes unstable and chaotic at this value of the Weissenberg number. As the Weissenberg number further increases to 3, the vortices become more distorted, and the flow field becomes more chaotic, as can be seen from Fig. 2d. The fluctuating and chaotic nature of the flow field inside the microchannel is more evident in Fig. 3a, wherein the temporal variation of the non-dimensional stream-wise velocity component (obtained at a probe location placed at the origin) is plotted against the time both for Newtonian and viscoelastic fluids with various Weissenberg numbers. From this figure, it can be clearly seen that the velocity reaches a steady value with time both for Newtonian and viscoelastic fluids with $Wi =1$, thereby suggesting the presence of a steady flow field inside the microchannel. However, as the Weissenberg number increases to 2, the stream-wise velocity shows a fluctuating behaviour. It becomes more intensified as the Weissenberg number further increases to 3. Therefore, it clearly suggests that the flow field inside the microchannel becomes unsteady and chaotic at these values of the Weissenberg number.

To analyze the nature of this unsteadiness in the flow field, we have presented the power spectral density plot of these velocity fluctuations in Fig. 3b. The excitation of the fluid motion over a wide range of continuum frequencies can be seen from this Figure. This is one of the characteristic features of the elastic turbulence^21,28^. The amplitude of the power spectrum is higher at $Wi = 3$ than that obtained at $Wi = 2$. Therefore, it suggests that the intensity of the velocity fluctuations gradually increases as the Weissenberg number progressively increases. A plateau in the power spectrum is seen in the low-frequency range. Whereas, at high frequencies, a power-law decay $(\omega ^{\alpha })$ is seen in the power spectrum, which covers almost a decade of the frequency range. The fitted values of the power-law exponent $\alpha$ are –3.16 and –3.78 for Weissenberg numbers 2 and 3, respectively. The range of values of the power-law exponent seen in the present study is the same as that seen in the elastic turbulence originated in various pressure-driven flows^21,28^. All these suggest that a turbulence-like flow field is generated inside the microchannel, which is purely driven by the elastic stresses of the viscoelastic fluid. A discussion on this electro-elastic turbulence is presented in the subsequent section in detail.

**Figure 4:**
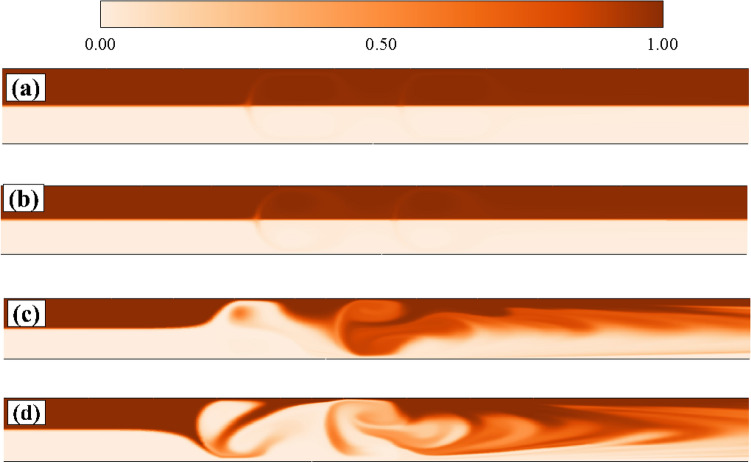
Instantaneous evaluation of the dye concentration both for Newtonian (**a**) and viscoelastic fluids at (**b**) Wi = 1 (**b**) Wi = 2 (**c**) Wi = 3.

Therefore, one would expect this locally generated electro-elastic turbulence to increase the mixing of two fluids even in this straight microchannel. To show this, we have used the same fluid which fills up the whole microchannel instead of using two different fluids. The fluid in the upper half of the microchannel is mixed with a dye of finite concentration, whereas the fluid in the lower half has a zero dye concentration. This will allow us to directly visualize the mixing phenomena of the fluids present in the upper and lower halves of the microchannel. The governing equation to track the evaluation of the dye concentration inside the microchannel is written in the preceding section. The results are presented in Fig. 4. For Newtonian fluids, it is found that the fluids that are present in the two halves of the microchannel are not mixed up, Fig. 4a. A similar pattern is also seen for viscoelastic fluids with low Weissenberg numbers, for instance, see the results at $Wi = 1$ in Fig. 4b. This is due to the absence of elastic instability and elastic turbulence at this Weissenberg number. However, as the Weissenberg number further increases to 2 (Fig. 4c), one can see the mixing of dye, particularly at the downstream section of the microchannel. It is thereby suggesting that the fluids which are present in the two halves of the microchannel are also mixed up. To quantitatively show the mixing efficiency of the two fluids, we have calculated the mixing index $\eta$ defined as^35,36^

10 $$\begin{aligned} \eta = 1 - \frac{\sqrt{\frac{1}{N}\sum _{1}^{N}({\bar{C}}_{s} - {\bar{C}}_{s}^{*})^{2}}}{\sqrt{\frac{1}{N}\sum _{1}^{N}({\bar{C}}_{s}^{0} - {\bar{C}}_{s}^{*})^{2}}} \end{aligned}$$

**Figure 5:**
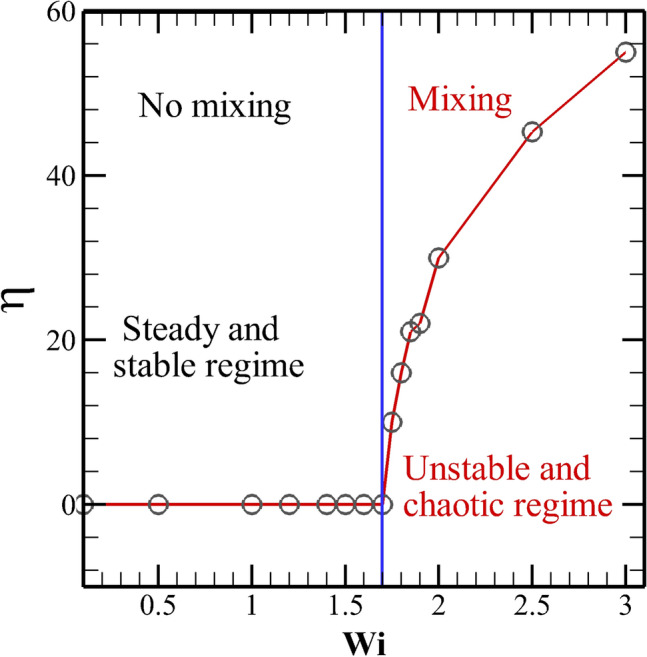
Variation of the mixing index $\eta$ with the Weissenberg number.

In the above equation, ${\bar{C}}_{s}$, $\bar{C_{s}^{*}}$ and $\bar{C_{s}^{0}}$ are the dye concentration at a point in the domain, dye concentration for a perfectly mixed fluid and dye concentration for an unmixed fluid, respectively. The value of $\bar{C_{s}^{0}}$ can be either 0 or 1, and hence the value of $\bar{C_{s}^{*}}$ would be 0.5. Therefore, the denominator of Eq. (10) has a constant value of 0.5. The theoretical range of $\eta$ lies in between 0 and 1, representing perfectly unmixed and mixed fluids, respectively. Note that the calculation of this parameter is performed at the exit of the microchannel. Figure 5 depicts the variation of $\eta$ with the Weissenberg number. From this figure, we can clearly observe that the value of $\eta$ remains zero up to a value of the Weissenberg number of around 1.7, thereby indicating that no mixing of fluids happens up to this value of the Weissenberg number. This is because of the fact that up to this critical value of *Wi*, the flow remains steady and no electro-elastic instability is seen to present in the domain that can promote the mixing. As the Weissenberg number is further incremented beyond this critical value, $\eta$ starts to attain a finite value which again gradually increases with the Weissenberg number. Therefore, the mixing of the fluids starts to happen after this critical value of the Weissenberg number due to the emergence of the electro-elastic instability and then electro-elastic turbulence on further increasing the Weissenberg number.

## Discussion

As mentioned in the previous section, the enhancement in the mixing phenomena of viscoelastic fluids in a straight microchannel is due to the presence of local chaotic and fluctuating flow field caused by the electro-elastic turbulence in the system. Before the transition to this chaotic flow regime, an electro-elastic instability should emerge in the system, which is the precursor of this turbulent flow regime. These elastic instabilities in viscoelastic fluids result from the interaction between the streamline curvature present in the system and the elastic stresses originated due to the stretching of viscoelastic microstructure. McKinley and co-workers established a criteria, named the Pakdel-McKinley criteria^23,42^, which is often used to explain these instabilities purely driven by the elastic forces. It is written as below

11 $$\begin{aligned} M = \sqrt{\frac{\tau _{11}}{\eta _{0} {\dot{\gamma }}} \frac{\lambda U}{{\mathcal {R}}}} \ge M_{crit} \end{aligned}$$

**Figure 6:**
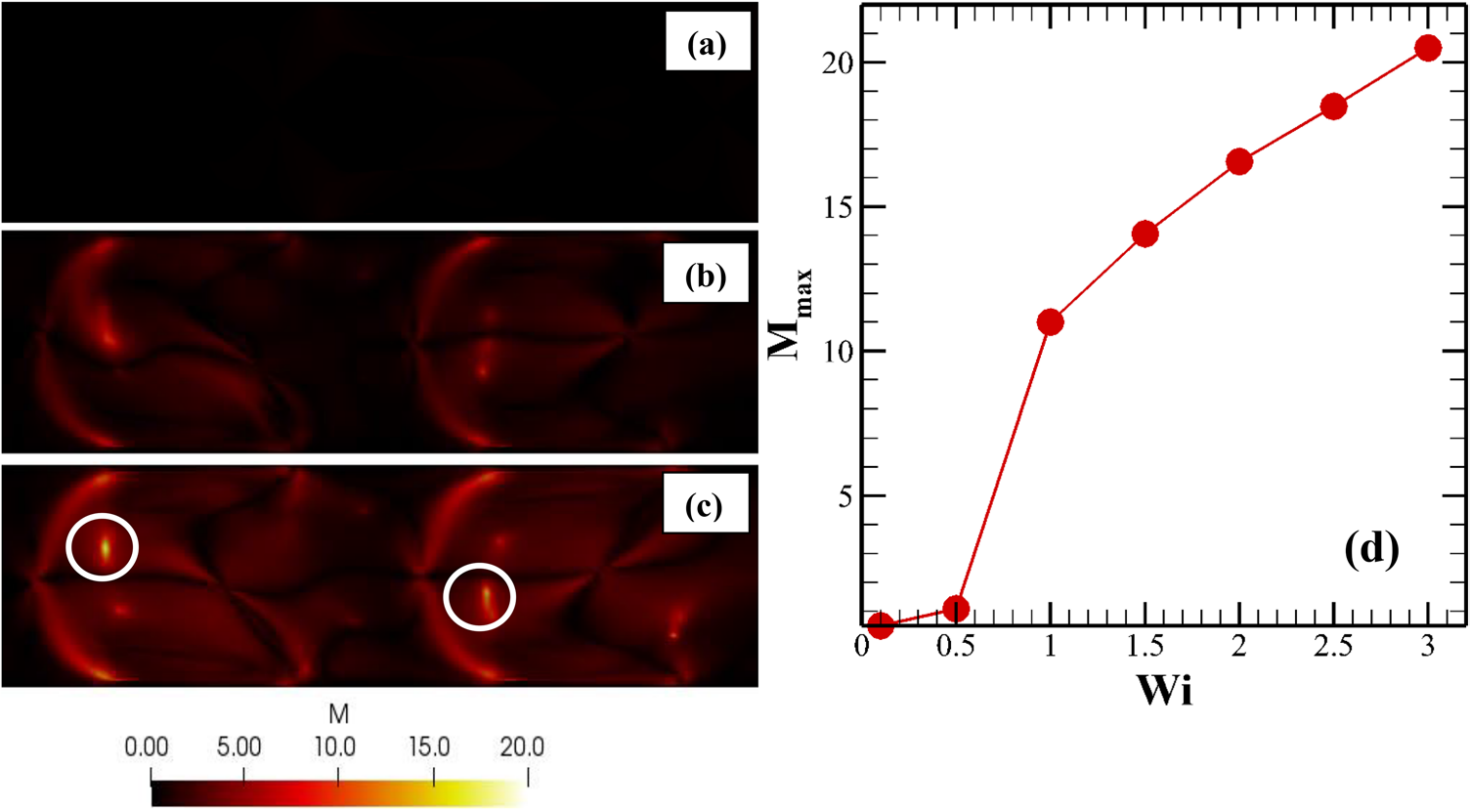
Surface plot of the distribution of the Pakdel-McKinley M parameter at (**a**) Wi = 0.1 (**b**) Wi = 1 and (**c**) Wi = 3. (**d**) Variation of the maximum value of the M parameter with the Weissenberg number.

In the above equation, $\tau _{11}$ is the normal stress in the flow direction along a streamline, ${\dot{\gamma }}$ is the characteristic value of the local deformation rate, ${\mathcal {R}}$ is the characteristic radius of the streamline curvature. For a two-dimensional flow field, one can determine these different parameters as follows^43^. The streamline curvature is calculated as $\frac{1}{{\mathcal {R}}(x, y)} = \frac{\left( \frac{\partial \psi }{\partial x}\right) ^{2} \frac{\partial ^{2} \psi }{\partial y^{2}} + \left( \frac{\partial \psi }{\partial y}\right) ^{2} \frac{\partial ^{2} \psi }{\partial x^{2}} - 2 \frac{\partial \psi }{\partial y} \frac{\partial \psi }{\partial x} \frac{\partial ^{2} \psi }{\partial x \partial y}}{\left[ \left( \frac{\partial \psi }{\partial x}\right) ^{2} + \left( \frac{\partial \psi }{\partial y}\right) ^{2} \right] ^{3/2}}$ where $\psi$ is the stream function and $u_{x} = -\frac{\partial \psi }{\partial y}$ and $u_{y} = \frac{\partial \psi }{\partial x}$. The normal stress along a streamline can be calculated as $\tau _{11} = {\mathbf {t}} \cdot \tau \cdot {\mathbf {t}} = \tau _{xx} t_{x}^{2} + \tau _{yy} t_{y}^{2} + 2\tau _{xy} t_{x} t_{y}$ where ${\mathbf {t}} = t_{x} e_{x} + t_{y} e_{y}$ is the tangent vector along a streamline whose components are calculated as $t_{x} = - \frac{\frac{\partial \psi }{ \partial y}}{|\nabla \psi |}$ and $t_{y} = \frac{\frac{\partial \psi }{ \partial x}}{|\nabla \psi |}$. Here $e_{x}$ and $e_y$ are the unit vectors in x and y directions, respectively. According to this criteria, when this non-dimensional parameter *M* exceeds a critical value $M_{crit}$, an elastic instability will emerge in the system. In this study, we have also calculated this criteria and plotted it in Fig. 6 for various values of the Weissenberg number. At low values of the Weissenberg number, for instance, at $Wi = 0.1$, the maximum M value was found to be around 0.52. This value is very small as compared to the values obtained for the onset of these instabilities for the various pressure-driven flows, for instance, for the flow past a cylinder, the critical value of M was found to be 6.2^42^, whereas for the flow of viscoelastic Boger fluids in a lid-driven cavity, it was between 3 and 4^44^. Therefore, at this low value of *Wi*, there is no elastic instability observed. However, as the Weissenberg number gradually increases to higher values, the M value also increases, for instance, see the results presented in Fig. 6 at $Wi = 3$. Note that here the M values at different Weissenberg numbers are presented on a scale of 0 to 20 to show a quantitative difference in its distribution obtained at different Weissenberg numbers. The M value at $Wi = 0.1$ is so small that it seems almost zero. At $Wi = 3$, we can see the presence of some small regions (marked with open circles in Fig. 6) where the maximum value of M is observed. These regions are most susceptible to the generation of these elastic instabilities and elastic turbulence. The variation of the maximum M values with the Weissenberg is shown in Fig. 6d. It is clearly seen that after a critical value of the Weissenberg number, the M value starts to increase rapidly, and the instability starts to appear when it reaches a critical value at a critical value of the Weissenberg number, as schematically shown in Fig. 6. On further increasing the Weissenberg number, these instabilities ultimately transit to the elastic turbulence regime with more chaotic flow behaviour, thereby promoting the mixing of two fluids.

It should be mentioned here that some earlier studies, where such electro-elastic instabilities were seen, did not find any significant improvement or even found reduction in the mixing of the fluids as compared to that seen for non-viscoelastic fluids. For instance, Bryce and Freemann^45^ carried out experiments with viscoelastic polyacrylamide polymer solutions flowing through a 2:1 micro constriction. Although they found the existence of the electro-elastic instabilities in the system once the applied electric field exceeds a critical value; however, they observed a reduction in the mixing phenomena compared to that seen only in the solvent (water + methanol) with no polymer additives. Furthermore, Pimenta and Alves^18^ also noticed no significant improvement in the mixing phenomena in both the cross-slot and flow-focusing devices based on these electro-elastic instabilities. This is in contrast to that seen in the present study, where we can clearly see the improvement in the mixing phenomena utilizing these electro-elastic instabilities and elastic turbulence. The possible reason for not showing the enhancement in the mixing phenomena of studies by Bryce and Freemann^45^ and Pimenta and Alves^18^ is that the elastic turbulence might not be developed to that extent which could ultimately lead to a greater mixing process. Therefore, the simple setup proposed in this study becomes more novel and relevant for electrokinetic mixing of viscoelastic fluids than those presented in earlier studies, such as cross-slot or micro constriction. Our proposed setup facilitates the crossing of streamlines (as shown and discussed in the previous section) through the horizontal mid-plane of the microchannel, i.e., at the interface of the two fluids. This, in turn, promotes a greater mixing phenomena by this chaotic convection. This was probably missing in earlier geometries, and hence the mixing was not achieved significantly.

## Conclusions

This study presents a very simple yet efficient approach for electrokinetic mixing of viscoelastic fluids in micro-scale systems based on extensive numerical simulations. Our proposed setup consists of a straight microchannel with patches of constant wall zeta potential that are present both on the top and bottom walls of the microchannel. The heterogeneity in the wall zeta potential at the microchannel wall creates an electro-elastic instability, which ultimately transits to the electro-elastic turbulence regime as the Weissenberg number is gradually increased. These locally generated instabilities and turbulence lead to the origin of a chaotic and fluctuating flow field, thereby promoting the mixing of viscoelastic fluids. Although some earlier investigations found the existence of such electro-elastic instabilities in electrokinetic flows of viscoelastic fluids; however, they did not observe any significant improvement^18^ or even found reduction in mixing of these fluids^45^ in geometries like a cross-slot cell or a flow-focusing device. However, in this study, our proposed setup (a straight microchannel with heterogeneous wall zeta potential) is not only relatively simpler as compared to the geometries like a cross-slot cell or flow-focusing device, but also shows a significant mixing of these viscoelastic fluids. We have carried out this study for fixed values of the applied voltage, wall zeta potential, polymer viscosity ratio, ion concentration, and a single arrangement of the wall patches. However, all these parameters may significantly influence the onset of these electro-elastic instabilities and elastic turbulence, and hence the mixing phenomena. We aim to investigate these in our future studies. Furthermore, the present study is totally based on numerical simulations, and therefore, it would be interesting to validate the efficiency of the proposed design by performing some corresponding experiments. In earlier experiments, more complex patterns for the surface wall zeta potential were created using the soft lithography technique^46^ than that proposed in the present study. Hence, we believe that the present setup, i.e., a straight microchannel with patches of finite wall zeta potential can be easily fabricated using the same soft lithography technique if one wants to perform the corresponding experiments or use it for a particular application.

## Methods

The present governing equations (Eqs. 1–9) have been solved using the finite-volume method based rheoEFoam solver available in the recently developed RheoTool package^47^. It has been developed based on the platform of an open-source computational fluid dynamics (CFD) code OpenFOAM^48^. A detailed description of the present solver used in this study is available elsewhere^18^, and hence only some of the salient features are recapitulated here. All the advective terms in the governing equations were discretized using the high-resolution CUBISTA (Convergent and Universally Bounded Interpolation Scheme for Treatment of Advection) scheme for its improved iterative convergence properties. All the diffusion terms in the governing equations were discretized using the second-order accurate Gauss linear orthogonal interpolation scheme. All the gradient terms were discretized using the Gauss linear interpolation scheme. The Euler time integration scheme was used to discretize the time derivative terms. While the linear systems of the pressure, velocity and electric potential fields were solved using the Preconditioned Conjugate Solver (PCG) with DIC (Diagonal-based Incomplete Cholesky) preconditioner, the stress and dye concentration fields were solved using the Preconditioned Bi-conjugate Gradient Solver (PBiCG) solver with DILU (Diagonal-based Incomplete LU) preconditioner. The pressure-velocity coupling was accomplished using the SIMPLE method, and the log-conformation tensor approach was used to stabilize the numerical solution. Furthermore, the relative tolerance level for the pressure, velocity, stress, and concentration fields was set as 10$^{-10}$. The creation of domain and meshing of it with regular hexahedral cells was performed using the blockMeshDict subroutine available in OpenFOAM.

The boundary conditions employed in this study are as follows: for the velocity, a zero gradient $(\nabla \mathbf{u } = 0)$ at the channel inlet and outlet and a no-slip condition $(\mathbf{u } = 0)$ at the channel wall; for the pressure, a fixed zero value $(p = 0)$ at the channel inlet and outlet and a zero gradient $(\nabla p = 0)$ at the channel wall; for the electric potential, a zero gradient $(\nabla \psi = 0)$ at the channel inlet and outlet, a fixed negative value $(\psi = - \zeta_{0} )$ on the wall patches and a zero value $(\psi = 0)$ on the rest of the channel walls; for the viscoelastic stress, a zero gradient $(\nabla \tau = 0)$ at the channel inlet and outlet whereas the values are linearly extrapolated on the channel walls; for the dye concentration, a fixed positive value $(c = 1)$ at the channel upper half and a zero value $(c = 0)$ at the lower half, a zero gradient $(\nabla c = 0)$ at the outlet and at the channel walls.

**Figure 7:**
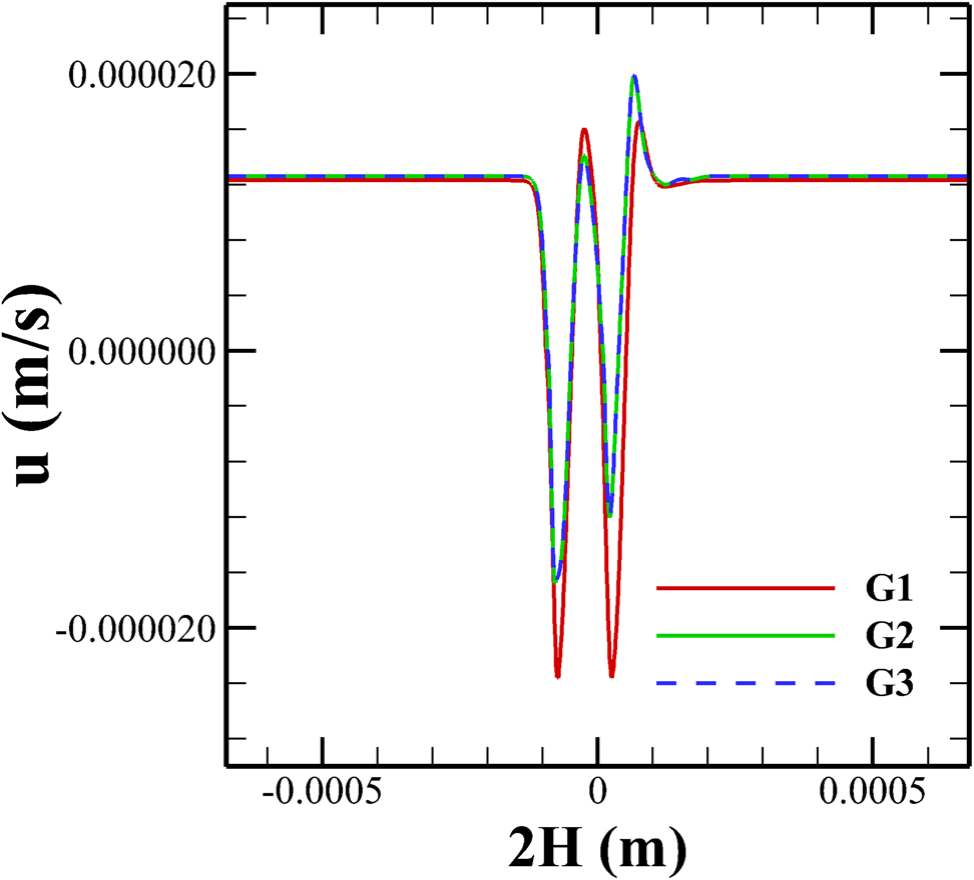
Grid independence study.

After fixing different discretization schemes for different terms of the governing equations and employing proper boundary conditions, we next turn our attention to choose an optimal grid density based on the standard procedure of the grid independence study. In doing so, three different grids with a different number of total hexahedral cells, namely, G1 (11680), G2 (49600) and G3 (99200) were created, and the simulations were run at the highest value of the Weissenberg number considered in this study, i.e., at $Wi = 3$. After obtaining the results at different grid densities, the comparison was performed in terms of the variation of the time-averaged stream-wise velocity component along the horizontal mid-plane passing through the origin, see Fig. 7. It can be seen the results become almost indistinguishable from each other as one moves from grid G2 to G3. Therefore, grid G2 was found to be suitable to carry out the present study. Along with the velocity component, the stress fields obtained with different grid densities were also compared (not shown here), and once again, the grid G2 was found to be the optimum one, which at the one hand, didn’t demand excessive computational resources and on the other hand, it also didn’t compromise with the accuracy of the present numerical results. Likewise the grid independence study, we have also carried out a systematic time independent study in order to choose an optimal time step size to carry out the present unsteady flow simulations. By performing so, a non-dimensional time step size of $\Delta t =$ 0.0001 was found to be sufficient for the present study.

**Figure 8:**
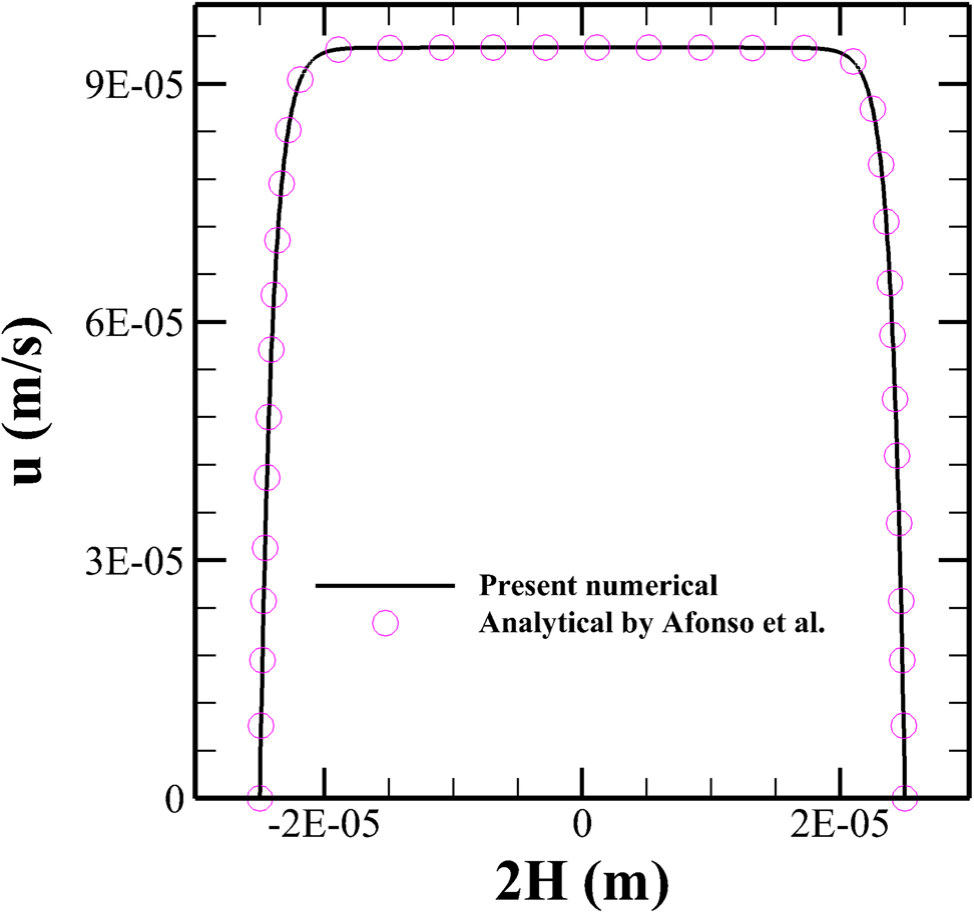
Comparison of the stream-wise velocity in a straight microchannel with uniformal wall zeta potential between the present (line) and analytical (symbols) results of Afonso et al.^49^.

**Figure 9:**
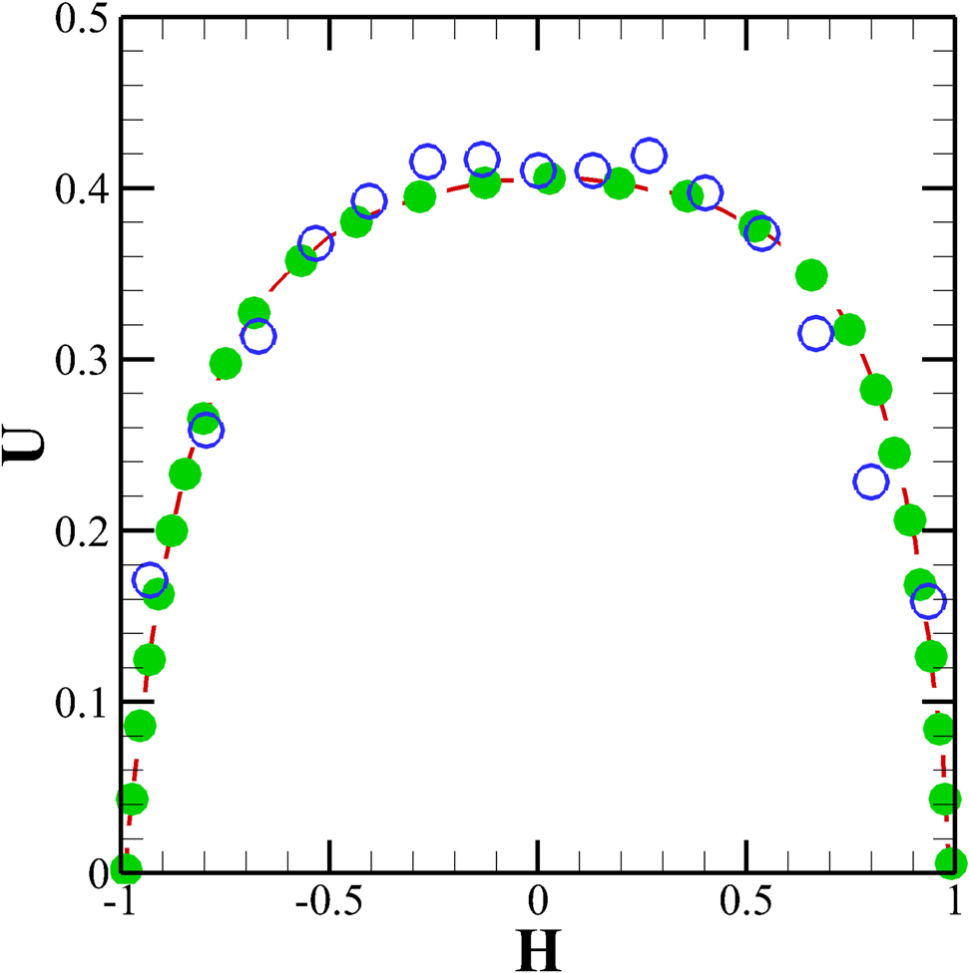
Comparison of the stream-wise velocity in the grooved area among the present (line) and numerical (filled symbols) and experimental results (open symbols) of Kim et al.^50^. Note that here the velocity and distance are presented in non-dimensional form as that used by Kim et al.^50^.

**Figure 10:**
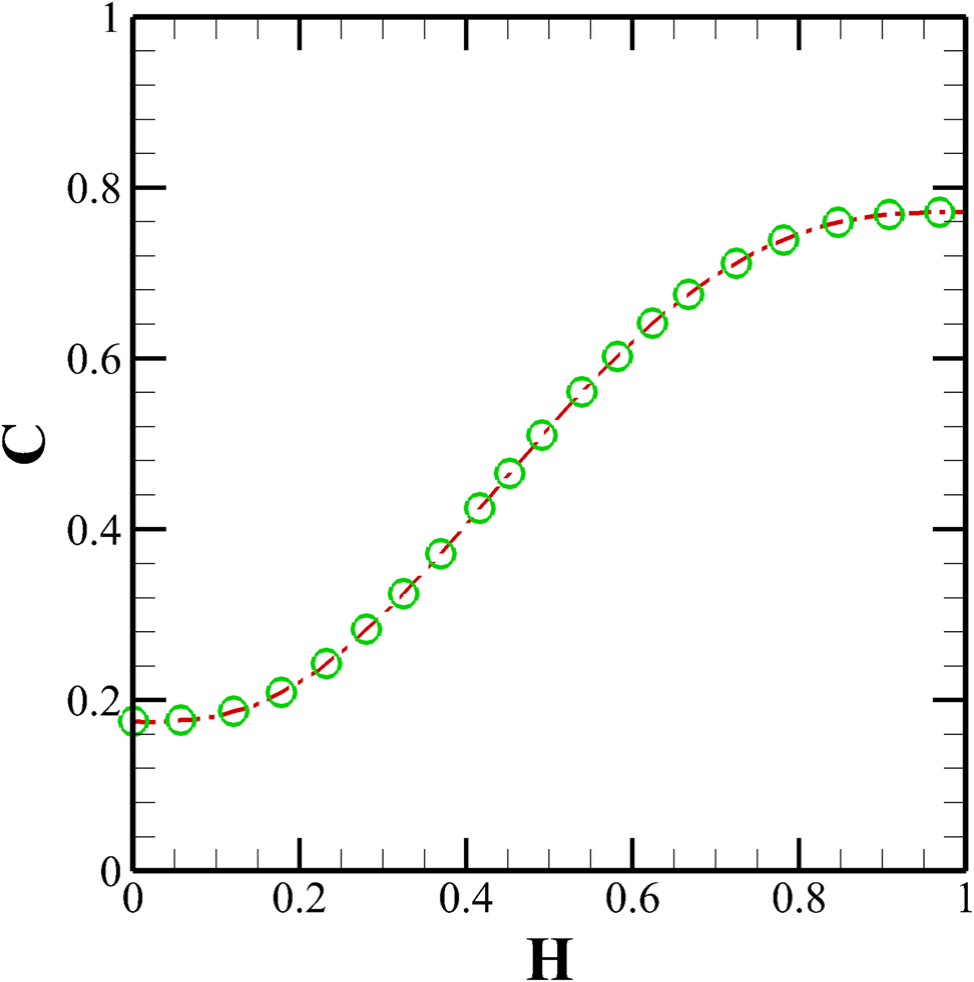
Comparison of the species concentration profile at the outlet of a straight microchannel between the present (line) and numerical results (symbols) of Hadigol et al.^51^.

Finally, we have carried out some validation studies in order to establish the accuracy and reliability of the present numerical setup that we have used in this study. Figure 8 shows the comparison between the present results with that of analytical results of Afonso et al.^49^ in terms of the variation of the stream-wise velocity in a straight microchannel with uniform wall potential for Oldroyd-B viscoelastic fluids. An excellent agreement can be seen between the two results. To gain more confidence in the current numerical settings, we have presented a validation with an experimental study performed by Kim et al.^50^ on the electrokinetic flow of Newtonian fluids in a microchannel with grooved surface, Fig 9. One can see a very good match with their numerical results and a reasonable good agreement with the corresponding experimental results. To further validate the implementation of the species transport equation, we have presented some validation in terms of the variation of the species concentration at the channel outlet between the present results with that of Hadigol et al.^51^ for the electrokinetic flow of Newtonian fluids in Fig. 10. Once again, a very good match can be seen between the two results.
